# How well supported are employees with terminal illness in the workplace? A snapshot of evidence from the Higher Education sector

**DOI:** 10.1177/02692163251393584

**Published:** 2025-11-08

**Authors:** Rehaan Husain, Joanne Smithson, India Tunnard, Michele Rose, Rachel Suff, Izzie Baverstock-Poppy, Sabrina Bajwah, Joanna M. Davies

**Affiliations:** 1 Cicely Saunders Institute of Palliative Care, Policy and Rehabilitation, Faculty of Nursing, Midwifery and Palliative Care, King’s College London, UK; 2 What Works Centre for Wellbeing, London, UK; 3 Open University, UK; 4 Chartered Institute for Personal Development, London, UK; 5 Marie Curie, London, UK; 6 Kings College Hospital NHS Foundation Trust, London, UK

**Keywords:** terminal illness, terminal care, occupational health, health policy, quality of life, social support, palliative care, workplace, personnel management, employment

## Background

Approximately 1 million people of working-age die in Europe each year.^1^ Compared to older populations, this cohort is more likely to face financial challenges related to loss of income, cost of childcare, and mortgage and rent costs.^2^ Many choose to continue working after a terminal diagnosis and employers have an important role in supporting people to remain in work and to leave with dignity when the time is right. Good support can include offering flexibility and adjustments to roles for example through job crafting,^3,4^ managing sickness absence outside of standard reporting systems, and providing an open and supportive culture.^5^ Yet, survey evidence from the UK has highlighted an overall lack of targeted support and an “employer lottery” with wide variation in support between employers.^2^

The university sector is a major employer across the globe; in the UK it employs over a quarter of a million people.^6^ To understand more about how staff with terminal illness are supported in the higher education sector, we undertook a review of policies across the 24 leading Russell group universities in the UK, to explore variation in provision and make recommendations for improvements. Our study focused on the research-intensive Russell Group universities in the UK as a barometer for circumstances in the rest of the sector.

## Methods

We obtained sickness policies and related documents from university websites and through Freedom of Information requests. We extracted data on key features of policies based on guidance for provisions for staff with terminal illness recently developed by the Chartered Institute for Personal Development (CIPD), the professional body for Human Resources in the UK.^7^ We present the data by institution and provide a summary of best practice examples.

## Results

We obtained data for all 24 universities. Table 1 provides a summary of key aspects of policies and Table 2 highlights examples of best practice.

**Table 1.** A summary of sickness and terminal illness policies at UK research-intensive universities.

University	Sickness policy/ies mentions long-term illness	Sickness policy/ies mention terminal illness	Maximum sick pay entitlement (number of months on full pay, number of months on half pay)	Years of service required to be eligible for the maximum sick pay entitlement	Has written guidelines for handling a death in service (separate from pension processes)	Has signed the Dying to Work Charter^a^
Summary of results (out of 24 Universities in total)	*n* = 23	*n* = 8	6, 6: *n* = 22 8, 4: *n* = 1	5: *n* = 12 4: *n* = 4 3: *n* = 6 0: *n* = 1	*n* = 12	*n* = 1
University of Birmingham	Y	N	6, 6	0^b^	N	N
University of Bristol	Y	N	6, 6	5	Y	N
University of Cambridge	Y	Y	6, 6	5	N	N
Cardiff University	Y	N	No data^c^	No data^c^	N	N
Durham University	Y	N	6, 6	5	Y	N
University of Edinburgh	Y	Y^d^	6, 6	3	Y	N
University of Exeter	Y	N	6, 6	3	Y	N
University of Glasgow	Y	N	6, 6	3	N	N
Imperial College London	Y	Y	6, 6	4	Y	N
King’s College London	Y	N	6, 6	3	Y	N
University of Leeds	N	N	6, 6	5	Y	N
University of Liverpool	Y	Y^d^	6, 6	4	N	Y
London School of Economics	Y	N	6, 6	5	N	N
University of Manchester	Y	N	6, 6	3	N	N
Newcastle University	Y	N	6, 6	5	N	N
University of Nottingham	Y	N	6, 6	5	N	N
University of Oxford	Y	N	6, 6	5	Y	N
Queen Mary University	Y	Y^d^	6, 6	3	Y	N
Queen’s University of Belfast	Y	Y^d^	6, 6	5	N	N
University of Sheffield	Y	N	6, 6	5	Y	N
University of Southampton	Y	N	6, 6	5	Y	N
University College London	Y	N	6, 6	4	Y	N
University of Warwick	Y	Y	6, 6	4	N	N
University of York	Y	Y^d^	8, 4	5	N	N

a The Dying to Work Charter is a voluntary charter which aims to protect the rights of terminally ill employees. It outlines an agreement that supports, guides and safeguards employees who have a terminal diagnosis. https://www.dyingtowork.co.uk/.

b Birmingham University have “day one rights,” for all staff groups, meaning no length of service is required to be eligible for the maximum sick pay entitlement.

c Cardiff University did not provide data on sick pay entitlements.

d These universities had a specific policy regarding terminal illness.

**Table 2.** Best practice examples from sickness or terminal illness policies at research-intensive universities, aligned with Chartered Institute for Personal Development (CIPD) guidelines.

Category^a^	Summary of CIPD guidelines	Examples of best practice from sickness or terminal illness policies
Developing a framework to support employees	Policies should include explicit provisions for supporting staff with terminal illnesses and cover the following key policy areas: a statement of principles, policy objectives (key actions and outcomes), key responsibilities for each department, managing and recording absences, pay and time off, benefits and health insurance, internal and external support, mental health support, bereavement support, and activities or initiatives to support terminally ill employees.	Five universities had a dedicated terminal illness policy. Liverpool University’s terminal illness policy was one of the most comprehensive and detailed the roles of Human Resources, Occupational Health, and line managers in supporting employees with terminal illnesses. This included reference to reasonable adjustments, support for ill-health retirement, Employee Assistance Programs (EAPs) that provide counselling and financial advice, details of guidance and training available to line managers, mention of the university’s commitment to the Trades Union Congress (TUC) Dying to Work Charter, and a written commitment to making employees aware that they will not be dismissed as a result of their condition. Queen Mary University of London’s terminal illness policy mentioned the consideration of extending periods of sick pay or reinstating full pay for terminally ill employees. It also detailed access to independent financial advice and assistance in planning the remainder of their working life.
Creating an inclusive and supportive culture	Policies should help raise awareness and understanding among colleagues to build a compassionate work culture for terminally ill employees. Policy provisions must ensure employers communicate an empathetic and sensitive approach regarding terminal illness.	Within their terminal illness policies, some universities stated the importance of acknowledging the wishes of the terminally ill employee. For example, Queen Mary’s University’s terminal illness policy mentioned the importance of compassion and sensitive consideration when dealing with terminally ill employees. Edinburgh University’s terminal illness policy stated that managers would strive to take the views of a terminally ill employee into account when identifying ways to support them.
Occupational benefits and health insurance	Policies should offer effective financial support to employees with terminal illnesses. They should clearly outline information regarding the financial needs of employees with terminal illnesses.	Twelve universities included a detailed outline of proceedings for ill-health retirement in a dedicated section within their sickness policy. University College London’s sickness policy included a comprehensive description of the process of ill-health retirement, where to access further information, who to contact for further support, and pension scheme requirements. Only Liverpool University has signed up for the Trade Union Congress (TUC) Dying to Work Charter, which includes a pledge to protect death in service benefits (lump sum payments paid to loved ones following the death of an employee) for terminally ill employees. It also includes a pledge that they will not dismiss terminally ill staff due to their diagnosis. It is important to note that a pledge of no redundancy may not be viable in safety critical roles. Furthermore, in some cases redundancy may be the desired or financially beneficial outcome for an employee.
Managing absence with compassion and flexibility	Policies must ensure that their absence management system is easy to navigate and enables managers to manage leave and sickness absence in an empathetic and supportive way. Employee absences related to terminal illness must be handled outside of normal reporting procedures and organisations should avoid a rigid approach to absence management.	Seven universities had sickness policies that clearly stated that absences for employees with terminal or long-term illnesses would be handled outside of normal reporting procedures. Queen Mary’s University’s sickness policy makes explicit reference to how employees with a terminal illness require a more flexible approach to the management of their attendance.
Promote good people management	Policies should ensure that line managers are confident and compassionate in managing employees with terminal illnesses. This may include access to training at the point of need, practical advice, and specific guidance in managing employees with terminal illnesses.	We found details from six universities about the training they offered to line managers in supporting employees with long-term illnesses. Notably, Liverpool University’s terminal illness policy explicitly mentioned training available for line managers in supporting staff with terminal illnesses. The University of Glasgow has a mandatory “Implementing Reasonable Adjustments” training course for all line managers.
Internal and external sources of information and support	This could include occupational health, Employee Assistance Programs (EAPs), counselling services, internal employee networks, signposting of external support groups and specialist charities, and relevant internal policies such as flexible working.	Twenty one universities outlined some examples of support mechanisms for employees within their sickness policies. Imperial College London’s provides a comprehensive example, signposting support within their main sickness policy that includes support from a dedicated Equality, Diversity and Inclusion Centre, buddy support for members of staff with a health condition, and counselling. The policy also detailed the roles of line managers, Human Resources, and Occupational Health in supporting employees with a phased return to work or redeployment and included case study examples regarding how to make physical and non-physical reasonable adjustments for employees. These policies facilitate job crafting, allowing terminally ill employees to adjust their roles and responsibilities to better suit their needs.

a Categories are based on the CIPD guidelines^7^ but are not a complete list.

## Discussion

In this review of the 24 research-intensive UK universities, we found that most (19) do not have special policy provisions for staff with terminal illness. While 23 mention long-term illness in their policies, only 8 refer to “terminal illness” and just 5 universities have a dedicated terminal illness policy. Despite an overall lack of dedicated support, we did find examples of best practice, including 7 universities that have a policy to manage absences outside of normal practices, 6 that signpost training for line managers, and 14 that provide clear examples regarding the implementation of reasonable adjustments.

A recent survey of over 1000 HR professionals^2^ found that 44% of organisations had policies in place for terminal illness and 43% of organisations managed absences for an employee with terminal illness outside of standard reporting processes. Comparatively, only 21% and 29% of Russell Group Universities had similar provisions, respectively, suggesting that the HEI sector may be lagging behind other employers in supporting staff with terminal illnesses.

The Dying to Work Charter is a voluntary charter which aims to protect the rights of terminally ill employees, including a pledge of no redundancy for terminally ill staff. Given that the higher education sector overall employs 33% of staff on fixed-term contracts,^8^ a commitment to no redundancy may be challenging to align with and could explain why only one university in the Russell Group has signed up to the Charter. Worryingly, none of the university policies we reviewed detailed specific provisions for fixed-term contract staff who are living with terminal or serious illness. For these staff, a lack of clear information about how the end of their contract will be handled is likely to exacerbate their financial insecurity and represents a serious gap in the current policy provision in this sector. Only one university offered “day one rights” for enhanced sick pay provisions. This contrasts with a growing trend towards offering “day one rights” for other provisions such as maternity pay, which is better aligned with a sector reliant on transient, fixed-term contract workers.^9^

Support for working-aged people living with terminal illness is an important and often neglected area of policy and provision in many countries across the globe. This study offers a snapshot of evidence about one sector of employment, revealing a lack of support and identifying ways to improve provision. Our review was limited by focussing only on Russell Group universities. It provides an indication of provision and support in the higher education sector but may miss examples of best practice in other universities. Our findings contribute to international evidence highlighting significant gaps and variation in support offered to employees living with a serious illness.^10^ We encourage universities to review their sickness policies in line with the CIPD guidance, and as part of this consider how practical approaches including absence management, role adjustments, and job crafting can be used to maximum effect. Universities should also ensure equality of provision for staff on fixed-term contracts who make up a large proportion of the higher education workforce.
